# Hydrogen Sulfide and Its Donors: Keys to Unlock the Chains of Nonalcoholic Fatty Liver Disease

**DOI:** 10.3390/ijms232012202

**Published:** 2022-10-13

**Authors:** Xianghui Li, Kaixin Jiang, Yantian Ruan, Siyuan Zhao, Yiming Zhao, Yuhua He, Zhili Wang, Jiacun Wei, Qiming Li, Changyong Yang, Yanzhang Li, Tieshan Teng

**Affiliations:** 1 School of Nursing and Health, Henan University, Kaifeng 475004, China; 2 Institute of Biomedical Informatics, School of Basic Medical Sciences, Henan University, Kaifeng 475004, China

**Keywords:** hydrogen sulfide, non-alcoholic fatty liver disease, H_2_S donor

## Abstract

Hydrogen sulfide (H_2_S) has emerged as the third “gasotransmitters” and has a crucial function in the diversity of physiological functions in mammals. In particular, H_2_S is considered indispensable in preventing the development of liver inflammation in the case of excessive caloric ingestion. Note that the concentration of endogenous H_2_S was usually low, making it difficult to discern the precise biological functions. Therefore, exogenous delivery of H_2_S is conducive to probe the physiological and pathological roles of this gas in cellular and animal studies. In this review, the production and metabolic pathways of H_2_S in vivo, the types of donors currently used for H_2_S release, and study evidence of H_2_S improvement effects on nonalcoholic fatty liver disease are systematically introduced.

## 1. Introduction

Hydrogen sulfide (H_2_S), well-known for its “rotten egg” odor, has been thoroughly described as a deadly toxic gas for centuries [1,2]. However, H_2_S has also been recognized as the third gaseous signaling molecule besides carbon monoxide (CO) and nitric oxide (NO) [3,4], improving various pathological processes, including angiogenesis, neuromodulation, inflammation, apoptosis, and tumorigenesis. Paradoxically, H_2_S, on one hand, acts as a physiological intercellular messenger to improve the therapeutic effect in some diseases; on the other side, it shows cytotoxic activity at high concentrations above physiological levels. Since the toxic effect of H_2_S is beyond its physiological range as a gas transmitter, it will not be discussed in this article.

The liver is a vital organ in the production and metabolism of H_2_S. In the last decade, accumulating evidence has suggested the critical function of H_2_S in the occurrence and development of several liver diseases, such as drug-induced liver injury caused by acetaminophen, acute liver injury, ischemia-reperfusion, and liver cirrhosis [5,6,7,8,9]. Furthermore, numerous studies revealed that the endogenous production of H_2_S was impaired in high-fat diet (HFD)-fed mice with non-alcoholic-steatohepatitis (NASH).

Nonalcoholic fatty liver disease (NAFLD) is characterized by abnormal lipid accumulation in the liver of individuals who do not drink alcohol excessively [5]. NAFLD is a common form of chronic liver disease [6] that consists of four major stages: nonalcoholic fatty liver (NAFL), NASH, hepatic fibrosis (HF), and hepatic cirrhosis (HC) [7,8]. NAFLD can lead to hepatic failure, which is characterized by massive hepatocyte necrosis and hepatic dysfunction, and it has a high fatality rate [9,10]. Patients with NAFLD are at a several times higher risk of developing hepatocellular carcinoma (HCC) than the general population [11,12]. As one of the most common chronic liver diseases worldwide, NAFLD imposes a heavy physical burden on patients, as its shackles on people’s bodies and lives is similar to a chain [13,14]. Moreover, NAFLD imposes a severe financial burden on patients and their families [15]. This paper systematically introduces the generation and metabolic pathways of H_2_S in vivo, various types of donors currently used for H_2_S release, and study evidence of H_2_S improvement effects on NAFLD.

## 2. Generation and Metabolic Pathways of H_2_S

### 2.1. Metabolism and Production of H_2_S In Vivo

#### 2.1.1. Metabolism of H_2_S In Vivo

**Figure 1:**
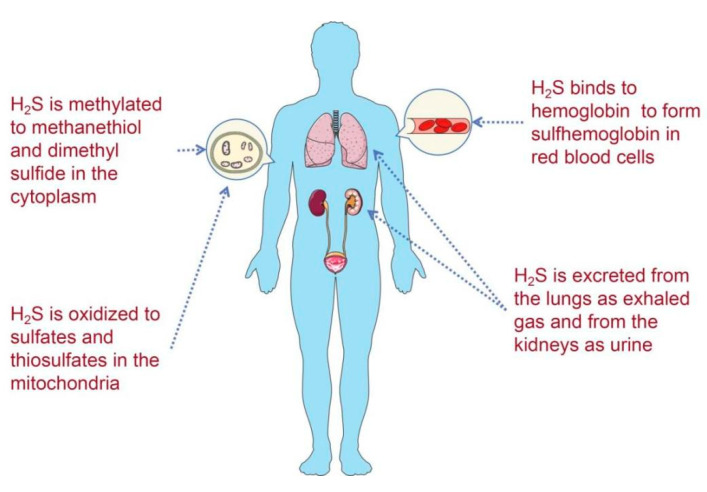
Four H_2_S decomposition pathways in mammals.

Unlike NO, H_2_S is relatively stable in body fluids. In the circulation or cytoplasm, free H_2_S can be scavenged by oxidation, methylation, and binding to methaemoglobin and excreted as gas or urine (Figure 1). To maintain the physiological balance of H_2_S to limit the adverse effects of H_2_S, four H_2_S decomposition pathways have been detected in mammals [16,17]. First, free H_2_S can be methylated to yield methanethiol (CH_3_-SH) and dimethylsulfide (DMS, CH_3_-S-CH_3_) by thiol S-methyltransferase in the cytoplasm [18]. Second, H_2_S is initially oxidized by sulphide quinone oxidoreductase (SQR), generating an SQR-bound persulfide intermediate. Then, the SQR-bound persulfide is transferred to an acceptor, such as GSH, yielding a molecule of oxidized glutathione (GSSG) in this process. Moreover, GSSH is converted to sulphite (SO_3_^2^^−^) by ethylmalonic encephalopathy protein 1 (ETHE1). The formed sulphite can either be converted into thiosulphate (S_2_O_3_^2^^−^) by thiosulphate sulphurtransferase, or it can be directly used by sulphite oxidase (SUOX) to generate sulphate (SO_4_^2^^−^) [19,20]. Third, H_2_S can be scavenged by methemoglobin, forming green sulfhemoglobin [20,21,22]. However, the mechanism of H_2_S binding to hemoglobin is not clear. Finally, these sulfur-containing substances can be excreted from the body as exhaled H_2_S gas or urine containing thiosulfate, sulfite, and sulfate [23].

#### 2.1.2. H_2_S Production In Vivo

Liver H_2_S is considered to be derived from endogenous liver synthesis and exogenous sources from the gastrointestinal tract. Exogenously, the gut microbiota is the major producer of H_2_S in vivo. In fact, the H_2_S level in sterile mice is 80% lower than in conventional mice [24]. L-cysteine can be metabolized in vivo to produce H_2_S through the cysteine desulphydrase from cysteine-desulfurizing bacteria, such as *E. coli* and *S. enterica* [25]. In addition, sulphate-reducing bacteria (SRB), including Desulfovibrio, Desulfobacter, and Desulfotomaculum, can also produce H_2_S via the reduction of inorganic sulphate or microbial catabolism of sulphomucins. As H_2_S is mainly produced from sulfur-containing amino acids, a high protein diet (HPD) can significantly change microbiota composition to increase the amount of H_2_S-producing bacteria [26]. In fact, several studies have revealed that mice fed with HPD exhibited an increase in sulphate-reducing bacterial abundance and higher amounts of colonic H_2_S [27]. Meanwhile, individuals fed with HPD for ten days had a 15-fold improvement in fecal sulfide compared with those fed with a vegetarian diet [28]. In other studies, Attene-Ramos et al. have shown in vitro that H_2_S in excess is detrimental for colonic epithelium energy metabolism and DNA integrity. However, intestinal cells can improve the H_2_S oxidation capacity in mitochondria to limit the adverse effects of H_2_S [29]. Furthermore, non- enzymatic H_2_S production pathways have been widely reported in mammals. Koj et al. reported that H_2_S was produced when rat liver mitochondria were incubated with oxygen, glutathione (GSH), and thiosulphate [30]. Studies have also demonstrated that coordinated catalysis of cysteine with ferric iron and vitamin B6 result in dose-dependent intra-vascular release of abundant H_2_S [31]. It has also been considered that a non-enzymatic H_2_S generation pathway may occur in the liver, which will hold high levels of iron storage.

**Figure 2:**
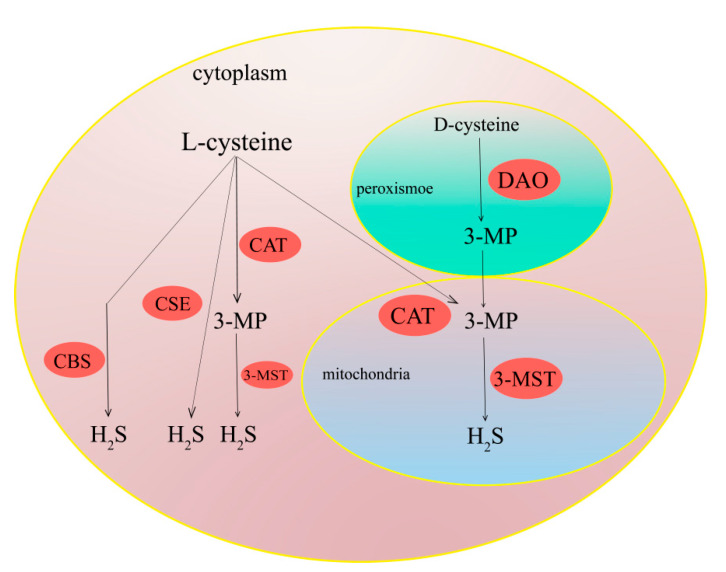
Pathway of H_2_S production in mammalian cells.

According to a number of recent studies, there are four enzymatic pathways to generate H_2_S in mammals, namely the cystathionine β-synthase (CBS) pathway, the cystathionine γ-lyase (CSE) pathway, the 3-mercaptopyruvate sulfurtransferase (3-MST)/cysteine aminotransferase (CAT) pathway, and the 3-MST/D-amino acid oxidase (DAO) pathway (Figure 2) [32,33,34]. CBS and CSE are the main enzymes that produce H_2_S in the liver, using L-cysteine, L-homocysteine, or L-cystathionine as the major substrates [35,36]. CBS is primarily expressed in the central nervous system and liver, while CSE is mainly located in the vascular system, liver, and kidney [37,38,39,40]. CBS, a unique heme-containing enzyme, can catalyze the pyridoxal-5-phosphate-dependent condensation of DL-homocysteine (DL-HCY) with serine to form L-cystathionine and water [41,42]. Then, L-cystathionine can be catalyzed by CSE to dimerize into L-cystine. L-cystine can be catalyzed by CSE or CBS via a β elimination reaction to yield H_2_S [43,44]. Moreover, 3-MST, along with CAT, is an important pathway for the production of H_2_S in mitochondria [45]. In the presence of CAT, L-cysteine can be catalyzed to transfer its amine group to α-ketoglutarate forming 3-mercaptopyruvate (3-MP) and glutamate. Then, 3-MP can be catalyzed by 3-MST to produce H_2_S [46,47]. It is worth mentioning that pyridoxal-5-phosphate is an indispensable cofactor for synthesizingH_2_S by CSE and CBS, while 3-MST requires zinc as a cofactor to synthesize H_2_S. Additionally, though CSE and CBS are mainly localized in the cytoplasm, they can translocate into mitochondria under certain oxidative conditions, whereas 3-MST usually reside and produce H_2_S in mitochondria. The DAO/3-MST pathway is the fourth H_2_S generation pathway in vivo, which was first discovered by Kimura et al. [32]. They found that kidney lysates can produce more than 60-fold H_2_S by using D-cysteine as a substrate compared with L-cysteine [48]. D-cysteine is oxidized to 3-MP, ammonia (NH_3_), and hydrogen peroxide (H_2_O_2_) in the presence of DAO. Then, 3-MP is introduced into mitochondria and metabolized by 3-MST to produce H2S. As DAO is only located in the brain and kidney, this H_2_S generation pathway is believed to exclusively exist in the above mentioned two organs [49].

### 2.2. Development of H_2_S-Based Therapeutics

Along with the demonstrated therapeutic effect, the development of H_2_S-based therapeutics relies on physiologically stable H_2_S donors, which can deliver H_2_S to the desired locations at the appropriate concentrations. Direct inhalation obviously would not be an acceptable approach for many reasons including smell, irritation, and enhanced local concentrations at the lung. More recently, an increasing number of H_2_S donors, as well as their H_2_S-related biological effects, have been reported [50]. In addition to natural H_2_S donors [51], more organic synthetic H_2_S donors have also been developed [52] (Table 1). In general, H_2_S donors can be classified as natural sulfur-containing organic compounds, inorganic sulfide salts, small-molecule synthetic organic compounds, and DTT (1, 2-dithiole-3-thiones)–coupled non-steroidal anti-inflammatory drugs (NSAIDs).

#### 2.2.1. Natural Sulfur-Containing Organic Compounds

Garlic and onions are recognized as the main source of natural H_2_S donors. Sulfur-containing organic compounds derived from garlic are generally byproducts of the breakdown of thiosulfinates (R-SO_2_-SR). Among them, Allicin, as the most common form of the thiosulfinates, can be decomposed into four different types of H_2_S-releasing compounds, namely diallyl sulfide (DAS), diallyl disulfide (DADS), S-allylcysteine (SAC), and diallyl trisulfide (DATS) [53,54]. The above-mentioned garlic-derived H_2_S donors can be converted into H_2_S by human red blood cells in the presence of naturally free thiols, such as homocysteine, GSH, *N*-acetylcysteine, and cysteine [55]. In cruciferous plants, the natural glucosinolates can be catalyzed by myrosinase to generate isothiocyanates (ITCs), which are H_2_S-releasing compounds, and they have preventive and therapeutic effects on various types of diseases. The limitations of these natural H_2_S donors are that they have poor water solubility and generate various byproducts after the H_2_S release [56].

#### 2.2.2. Inorganic Sulfide Salts

Inorganic sulfide salts, including CaS, Na_2_S, and NaHS, etc., have most widely been studied as H_2_S donors in medical studies [57,58,59,60]. Sulfide salts are generally solid analogs of the gas, which exist in the form of HS- anions and H_2_S molecules under the physiological condition. Na_2_S and NaHS (particularly NaHS) have been most commonly used to assess the therapeutic potential of exogenous H_2_S delivery, and CaS has been proven to be a more stable H_2_S donor reagent than Na_2_S and NaHS [61]. Physiological studies on H_2_S using sulfide salts usually need high-dose treatment, leading to a surge in H_2_S concentrations in the blood and tissues to physiological levels and then a rapid decline in H_2_S levels. This delivery strategy is significantly different from that of endogenous H_2_S generation in which concentrations are tightly regulated. These drawbacks make it necessary to explore novel H_2_S-donors continuously to control the dose, duration, timing, and location of H_2_S release.

#### 2.2.3. Lawesson’s Reagent

Lawesson’s reagent, a well-known H_2_S donor, can generate H_2_S in aqueous media over a considerably longer period than sulfide salts. However, Lawesson’s reagent has not been widely used as an H_2_S donor by researchers because of its poor water solubility. GYY4137, a high water-soluble derivative of Lawesson’s reagent, can generate H_2_S via hydrolysis [62]. Owing to its commercial availability and high water solubility, GYY4137 is the most extensively used H_2_S donor aside from sulfide itself. GYY4137 has been demonstrated to be a valuable H_2_S-releasing compound for researchers, particularly in a study of the effect of the H_2_S release rate on physiological outcomes. However, GYY4137 has an obvious disadvantage. It is usually prepared and sold as a dichloromethane complex, which is residual after crystallization. Dichloromethane can be metabolized again to produce CO, which is another gas signal molecule with a similar biological effect to that of H_2_S. Thus, the effects produced by GYY4137 may be attributed to CO [63,64].

#### 2.2.4. Dithiolthiones

1,2-Dithiole-3-thiones (DTTs) are a class of small-molecule synthetic organic H_2_S donors [65,66]. DTTs can be easily synthesized through the reaction of anethole with elemental sulfur. DTTs are commonly viewed as one of the hydrolysis-triggered H_2_S donors, which are capable of being easily linked to other molecules to prepare drug-DTT conjugates. The DTT moiety has usually been appended to non-steroidal anti-inflammatory drugs (NSAIDs) and studied rather extensively, such as HS-Aspirins, HS-Sulindac, HS-Naproxen, HS-Diclofenac, HS-Mesalamine (ATB-429), and HS-Indomethacin (ATB-43) [67]. However, two problems must be taken seriously when such compounds are used to investigate the biological function of H_2_S. First, it is not clear whether DTT can release sufficient H_2_S under physiological conditions. Second, anethole trithione (ADT), one of the DTT derivatives, is widely used for coupling other drugs to produce H_2_S-donating versions of these drugs. However, it is still unclear whether the biological function of ADT itself or the H_2_S released by ADT plays a role.

**Table 1:** Summary of current H_2_S donors.

H_2_S Donors	Chemical Compound	Bioactivity	Drawbacks	Ref.
Inorganic salts	NaHS/CaS/NaS_2_	Anti-inflammation, cardioprotective effects, diabetes amelioration	Action time short, uncontrollable	[68]
Lawesson’s reagent	GYY4137	Anti-inflammation, vasodilation	Slow hydrolysis rate, metabolized to CO	[62,69]
DTTs	ADT-OH	Reducing cell viability	Poor selectivity	[70]
	DTT-NSAID	Anti-inflammation	Increasing arterial pressure	[71]
Derivatives of Allium sativum extracts	DATS/DADS	Regulating blood vessels	Poor water solubility, generating byproducts	[72]
	SPRC	Anti-inflammation, anti-oxidation	Unstable and short half-life.	[55,73]
Derivatives of thioamino acids	Thioglycine/Thiovaline	Vasodilation	Poor selectivity, slow release rate	[74]
Derivatives of anti-inflammatory drugs	S-aspirin	Anti-inflammation, cardiovascular protection	Complications in the upper gastrointestinal tract	[71]
Derivatives of anti-inflammatory drugs	S-diclofenac	Anti-inflammation, gastrointestinal protection	High cardiovascular risk	[75]
Derivatives of anti-inflammatory drugs	ATB-429	Anti-inflammation	Increasing arterial pressure	[76]
Derivatives of anti-inflammatory drugs	ATB-346	Anti-inflammation, antipyretic, analgesic	Increasing arterial pressure	[77]
Thiol-triggered donors	*N*-Benzoylthiobenzamides	Cardioprotection	Poor selectivity	[78]
Thiol-triggered donors	Acyl perthiols	Cardioprotection	Poor selectivity	[79]
Thiol-triggered donors	Dithioperoxyanhydrides	Vasodilation	Poor selectivity	[80]
Thiol-triggered donors	Arylthioamides	Vasodilation	Poor selectivity	[81]
Thiol-triggered donors	S-Aroylthiooximes	Anti-cancer proliferation	Poor selectivity	[82]
Photosensitive H_2_S Donor	Geminal-dithiols	Restores anti-microbial resistance	Poor selectivity	[83]
Photosensitive H_2_S Donor	Ketoprofenate photocages	Unknown		[84]
Photosensitive H_2_S Donor	α-Thioetherketones	Anti-inflammation	Poor selectivity	[85]
Enzyme-triggered H_2_S donor	BW-HP-101	Esterase triggered, anti-inflammation	Unknown	[86]
pH-triggered H_2_S donor	JK-1/JK-2	MI/R protection	Unknown	[87]
Dual COS/H_2_S donor	*N*-Thiocarboxyanhydrides	Angiogenesis	Unknown	[88]
Dual COS/H_2_S donor	Arylboronate thiocarbamates	Cardioprotection	Unknown	[89]
Dual COS/H_2_S donor	o-Nitrobenzyl thiocarbamates	Unknown	Unknown	[90]

## 3. Association between H_2_S Level and NAFLD In Vivo

The dominant H_2_S generation enzymes in liver tissues depend on CSE rather than CBS and 3-MST enzymes. In NAFLD patients, the expression of hepatic CSE was significantly down-regulated by approximately 33% compared to that in non-NAFLD patients [91]. In line with the CSE down-regulation, the circulating cysteine and homocysteine were increased in NAFLD patients [92]. In mouse hepatocytes treated with OA, the production of H_2_S was reduced remarkably, accompanied by the formation of a large number of intracellular lipid droplets detected by lipid staining. Meanwhile, CSE expression was decreased significantly, but not the CBS and 3-MST enzymes [93]. In primary hepatocytes, CSE deficiency increased the formation of lipid droplets, which could be reversed by NaHS treatment.

In an HFD-induced NAFLD mouse, the CSE mRNA and protein expression was decreased by IHC staining [94,95]. In keeping with the CSE down-regulation, the hepatic H_2_S generation of the NAFLD mouse was also reduced. The H_2_S donor treatment, NaHS and GYY4137, dramatically attenuated HFD-induced steatosis by HE staining, as well as lowered the liver triglyceride level and cholesterol level [96]. Similarly, methionine-choline-deficient (MCD)-induced damage to NASH rats can be prevented with the treatment of H_2_S and its release agents [97]. Coinciding with the aforementioned changes, the down-regulation of fatty acid de novo synthesis associated genes (SREBP-IC, ACC, FAS, and SCD-1) was detected [98].

In the hepatocyte-specific CSE deletion mouse model was observed an approximately 75% decrease in the H_2_S generation, along with a relatively severe hepatic steatosis development [99]. Accordingly, the triglyceride and total cholesterol level was increased in CSE-knockout mice. In contrast, an enhancement of the CSE activity can inhibit lipid accumulation in hepatocytes [100]. A combination of glucose tolerance tests, insulin tolerance tests, and pyruvate tolerance tests in CSE-knockout mice suggested that the CSE deficiency exacerbated glucose homeostasis and insulin resistance.

## 4. Physiological Mechanism of H_2_S in Alleviating NAFLD

The pathological processes of NAFLD are closely related to lipid metabolism, autophagy, endoplasmic reticulum stress, oxidative stress, inflammation, etc. Meanwhile, a few reports have indicated that H_2_S can alleviate NAFLD by regulating these pathological processes.

### 4.1. H_2_S Alleviates NAFLD by Activating Autophagy

**Figure 3:**
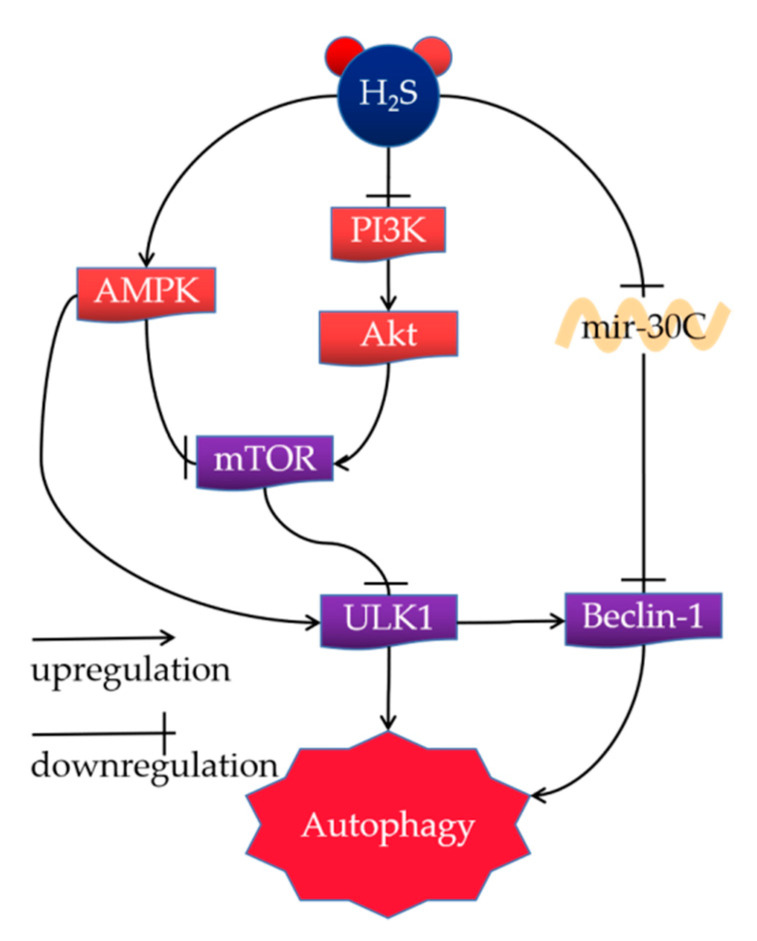
Mechanism of H_2_S regulation of autophagy in mammals.

Autophagy is a highly complex process of cellular degradation or organelle degradation. Dysfunctional autophagy is correlated with several diseases, including cancer, immune dysfunction, and NAFLD. It has been reported that H_2_S and its donors regulate autophagy through a few molecular mechanisms of alleviating NAFLD, such as the AMPK-mTOR pathway, PI3K/Akt/mTOR signaling pathway, and the Mir-30c signaling pathway (Figure 3).

#### 4.1.1. AMPK-mTOR Pathway

A potential mechanism of H_2_S ameliorating NAFLD was the simulation of liver autophagy by H_2_S through the AMPK-mTOR signal pathway [17]. AMPK is an essential initiator of autophagy, sensing ATP starvation, and cellular energy homeostasis. Its downstream regulatory proteins include the negative-regulation of the mTOR, whose down-regulation enhances autophagosome generation [101]. In contrast, suppression of the AMPK activation inhibits autophagy ability and results in the development of NAFL [102,103,104]. Similarly, knocking out AMPK in liver cell lines by siRNA blocks the pro-autophagy effect of NaHS. Mice treated with HFD generated a higher level of p-mTOR than those fed with a normal chow diet (NCD). In contrast, treatment with H_2_S reduced the phosphorylation and thus inhibited mTOR activation [105]. NaHS could decrease serum TG levels of HFD mice, which could be reversed via treatment with chloroquine (CQ), a well-known inhibitor of autophagy [106]. Moreover, NaSH enhanced the phosphorylation of AMPK and thus diminished the p-mTOR in a Western blot analysis. HFD treatment inhibited the phosphorylation of AMPK, which could be abolished through the co-administration of NaSH. Knock-down of the AMPKα2 subunit in mice inhibited the autophagic improvement effects of NaSH.

#### 4.1.2. PI3K/Akt/mTOR Signaling Pathway

The PI3K/Akt/mTOR signaling pathway is a vital pathway correlated with the regulation of autophagy by H_2_S [107]. It has been proven that H_2_S enhances autophagy by suppressing reactive oxygen species (ROS)-mediated PI3K/AKT/mTOR cascade in OA-induced LO2 cells [108]. In addition, NaHS and rapamycin significantly inhibit the protein expression of PI3K, Akt, and mTOR in HCC cells, indicating that the autophagy improvement by H_2_S is mainly initiated via the PI3K/AKT/mTOR signaling pathway. A number of studies have reported that high concentrations of H_2_S restrains the gene expression correlated with the PI3/Akt/mTOR pathway and increases the expression of other autophagy-related proteins, such as Beclin1, ATG5, and the ratio of LC3-II/LC3-I. It has been exhibited that inhibiting the PI3K/AKT signaling pathway via LY294002 reduces the improvement effect of H_2_S against scratch-induced cellular ROS level and NRF2 accumulation in the nucleus. The increased levels of autophagy in hepatocytes significantly enhance lipolysis and reduce the stress caused by fat accumulation in the liver [17,109].

#### 4.1.3. Mir-30c Signaling Pathway

Autophagy can also be induced by H_2_S via the mir-30c pathway [110]. Treatment with H_2_S can down-regulate mir-30c expression and up-regulate Beclin-1 and LC3 expressions. The in vitro experiments exhibited that mir-30c negatively regulated the Beclin-1 expression in cells by targeting the 3’UTR region. However, pre-incubation with the autophagy inhibitor 3-Methyladenine (3-MA) can eliminate the protective effect of H_2_S. These results suggest that H_2_S can play an autophagy role by inhibiting mir-30c and up-regulating Beclin-1 [110].

### 4.2. H_2_S Alleviates NAFLD by Regulating Inflammation

**Figure 4:**
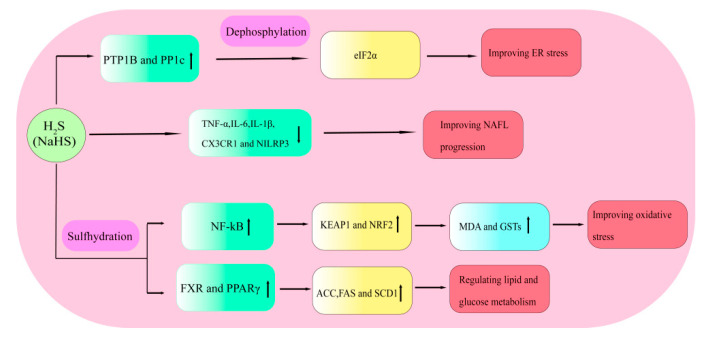
H_2_S alleviates NAFLD by improving inflammation, oxidative stress, lipid and glucose metabolism, and ER stress. Upward arrows indicate up-regulation of gene expression.

Numerous studies have demonstrated that H_2_S exerts various anti-inflammatory effects in tissues, including the liver tissues of patients with NAFLD. A previous study demonstrated that NaHS protected hepatocytes from PA-induced inflammatory damage through the down-regulation of the secretion of TNF-α, IL-6, IL-1β, and NLRP3. The LPS-induced RAW264.7 cells treated with NaHS can significantly decrease the protein expression of CX3CR1, an essential chemokine receptor in an inflammatory response (Figure 4). Meanwhile, accumulating evidences suggest that an over-expression of CSE can down-regulate the expression of CX3CR1 in IFN-γ-induced RAW264.7 cells [111]. Mice treated with NaHS suggest a decrease in the expression of CX3CL1 and hepatic TNF-α production, and an improving hepatic injury in the progression of NAFL [112]. Interference with CX3CR1 up-regulation inhibits the differentiation of moDCs, indicating that CX3CR1 may be served as a possible target for the therapy of NASH. However, contrary evidence shows that H_2_S exposure increases the expression of necrosis-related genes (RIPK1, RIPK3, MLKL, TAK1, and TAB3) and induces the TNF-α and IL-1β release, exhibiting an inflammatory response [113]. This inconsistency in the H_2_S treatment is attributed to the difference in H_2_S concentration.

### 4.3. H_2_S Alleviates NAFLD by Improving Oxidative Stress

Although the mechanism of oxidative stress in hepatocytes is particularly complex, it is generally characterized by increased ROS production and insufficient scavenging of ROS through endogenous antioxidant defense. Oxidative stress is an essential factor in the progression of NAFLD [98]. Therefore, regulating oxidative stress in hepatocytes is a potential strategy for treating NAFLD. Emerging data indicate that the palmitic acid-induced NAFLD cell model exhibits a high level of ROS by cell fluorescence detection. However, the over-expression of CES and CBS can decrease the ROS levels in a concentration-dependent manner. HFD feeding significantly boosts the generation of malondialdehyde (MDA), which is the final product of lipid peroxidation and serves as a biomarker of oxidative stress. A few studies have reported that an NaHS treatment can significantly reduce the formation of liver MDA and increase the activity of antioxidant enzymes [114]. Recent evidence has revealed that treatment with a low concentration of an H_2_S donor (NaHS or Na_2_S) can reduce lipid peroxidation levels in hepatocytes and increase the activity of antioxidant enzymes such as GSTs (Figure 4) [115]. It has also been demonstrated that H_2_S can inhibit the transcriptional activity of NF-κB, resulting in sulfhydration of Kelch-like ECH-associated protein 1 (KEAP1); the activated KEAP1 then releases active nuclear factor erythroid 2-related factor 2 (NRF2), causing an increased expression of antioxidant-response elements [116,117].

### 4.4. H_2_S Regulates Lipid and Glucose Metabolism

Several studies have proved that H_2_S can be severed as a crucial regulator of the hepatic lipid and glucose metabolism in NAFLD. The lack of endogenous H_2_S is the vital pathogenesis of dyslipidemia and hyperglycemia. Cai et al. found that H_2_S increases the triglyceride accumulation in mice fed with an HFD and weakens the insulin resistance of adipose tissues. Peroxisome proliferator-activated receptors γ (PPARγ) are ligand-activated nuclear receptors that regulate glucose and lipid metabolism. Endogenous H_2_S can enhance the PPARγ activity via the sulfhydration at the C139 site, thus increasing the glucose uptake and lipid storage in adipocytes [118]. Farnesoid X receptor (FXR), a type of nuclear receptor, plays an essential role in the pathological process of NAFLD by attenuating steatosis and enhancing insulin sensitivity [119]. Several studies have demonstrated that CSE knockdown can decrease the FXR mRNA and protein levels [91]. In contrast, overexpression of CSE or NaHS treatment can increase the FXR mRNA and protein levels. Furthermore, NaHS can promote the FXR sulfhydration at the Cys138/141 sites, thus increasing its activity to regulate the expression of the target genes correlated with glucose and lipid metabolism. These essential regulatory gene coding proteins consist of ACC, FAS and stearoyl-CoA desaturase 1 (SCD1) (Figure 4).

### 4.5. H_2_S Alleviates NAFLD by Improving ER Stress

The endoplasmic reticulum (ER) is a crucial organelle that provides a field for the folding and modifying of proteins. Excessive unfolded proteins in the ER will result in a situation called ER stress, which can be initiated through a series of internal or external environmental changes, including aging, environmental factors, and/or genetic mutations. Previous transcriptome data have indicated that the total protein expression in patients with NAFLD was significantly reduced compared to normal individuals [120]. The phosphatase PTP1B, located at the rough ER in the cytoplasm, plays an essential role in ER signaling. Several studies have demonstrated that a high endogenous H_2_S level can reduce ER stress by the sulfhydration of PTP1B [121,122]. The phosphorylation of eIF2α, leading to decreased protein synthesis, is a crucial biochemical step for ER stress. Yadav et al. have also reported that H_2_S can decrease the dephosphylation of the eukaryotic translation initiation factor 2α (eIF2α) via the sulfhydration of PP1c at Cys127and thus regulate the ER stress (Figure 4) [123,124].

## 5. Conclusions

In this work, we systematically described the pathway of H_2_S generation and metabolic pathways in vivo, the different types of H_2_S donors, and their effects on NAFLD. Accumulating evidence indicated that the H_2_S level was strikingly correlated with NAFLD in various physiological processes [125]. Whether the models were fatty acid-induced cell or high-fat diet induced animal, H_2_S undoubtedly exhibited its vital role in improving NAFLD. However, the stability and safety of conventional H_2_S donors could not meet the requirements of medical usage. Therefore, novel H_2_S donors with strong selectivity and safety remain required. The recent emergences of light-triggered H_2_S donors, pH-triggered H_2_S donors, and dual COS/H_2_S donors have provided a new strategy for the clinical application of H_2_S. Moreover, new mechanisms of H_2_S on improving NAFLD have been constantly updated in recent years. Increasing evidence indicates that modifying specific cysteine in target proteins via sulfhydration, including various enzymes and transcription factors, is an important mechanism for regulating the different pathological processes of NAFLD. 

## Data Availability

The study did not report any data.
